# Deep Ensemble learning and quantum machine learning approach for Alzheimer’s disease detection

**DOI:** 10.1038/s41598-024-61452-1

**Published:** 2024-06-20

**Authors:** Abebech Jenber Belay, Yelkal Mulualem Walle, Melaku Bitew Haile

**Affiliations:** Department of Information Technology, College of Informatics, University of Gondar, Gondar, Ethiopia

**Keywords:** Alzheimer’s disease, Deep learning, Ensemble model, Quantum machine learning classifiers, Neuroscience, Neurology

## Abstract

Alzheimer disease (AD) is among the most chronic neurodegenerative diseases that threaten global public health. The prevalence of Alzheimer disease and consequently the increased risk of spread all over the world pose a vital threat to human safekeeping. Early diagnosis of AD is a suitable action for timely intervention and medication, which may increase the prognosis and quality of life for affected individuals. Quantum computing provides a more efficient model for different disease classification tasks than classical machine learning approaches. The full potential of quantum computing is not applied to Alzheimer’s disease classification tasks as expected. In this study, we proposed an ensemble deep learning model based on quantum machine learning classifiers to classify Alzheimer’s disease. The Alzheimer’s disease Neuroimaging Initiative I and Alzheimer’s disease Neuroimaging Initiative II datasets are merged for the AD disease classification. We combined important features extracted based on the customized version of VGG16 and ResNet50 models from the merged images then feed these features to the Quantum Machine Learning classifier to classify them as non-demented, mild demented, moderate demented, and very mild demented. We evaluate the performance of our model by using six metrics; accuracy, the area under the curve, F1-score, precision, and recall. The result validates that the proposed model outperforms several state-of-the-art methods for detecting Alzheimer’s disease by registering an accuracy of 99.89 and 98.37 F1-score.

## Introduction

Alzheimer's disease (AD) is a degenerative brain disorder that diminishes memory and other critical mental abilities^1^. More broadly, dementia is described as the greatest global challenge for health care and social services, where globally approximately 50 million people were living with dementia in 2022^2^. AD is the most prevalent of dementia (60–70%), based on the study’s findings the estimation stated that over 150 million individuals will develop dementia by 2050^3,4^. AD and Dementia patients will face a variety of challenges, including cognitive impairment, memory loss, behavioral defects, difficulties with vision, and mobility issues that can render it difficult to do daily routine tasks^5^. It is a must to initiate treatment to slow down AD development in the early stages. To prevent the disease’s progression and lessen its long-term effects, it is crucial to investigate the early detection of Alzheimer's disease through specific Magnetic Resonance Imaging (MRI) scans of the brain. As an asymptomatic diagnostic procedure, structural MRI is accepted as a common imaging biomarker in identifying and categorizing AD stages as mild demented, moderate demented, very mild demented, and non-demented^6^. In light of the significant consequences of AD, clinicians have tried to identify patients who have mild memory loss that may progress into AD to intervene earlier^7^.

Deep learning techniques showed substantial improvement in disease prediction, detection, and classification tasks^8^. Ensemble Learning is one of the prominent techniques for boosting the performance of these deep learning models. It refers to the application of ensemble methods in which multiple Deep Learning (DL) models distinctly contribute to the detection and classification tasks^9^. Their outstanding prediction performance over their competent counterparts demonstrated them to be an effective addition to the field of predictive DL models. On another side, Quantum Machine Learning (QML), the intersection of classical machine learning and quantum computing, is a recently emerged field that has attracted scholars from a wide range of disciplines because of its flexibility, representation power, and promising scalability and speed results^10^. Various studies have confirmed that QML algorithms provide important advantages over traditional machine learning algorithms for many kinds of applications, including healthcare^11^.

Several medical sectors have adopted deep learning and machine learning approaches to predict, diagnose, and classify the possibility of Alzheimer's disease. However, the findings have been impacted by the shortage of data and the accuracy of the model. Making full use of a few resources to improve AD diagnostic accuracy poses a significant obstacle in boosting healthcare. Deep learning techniques are mostly employed to autonomously find patterns in the targeted dataset without the involvement of human intervention. The Ensemble Learning takes the benefit of two or more deep models to enhance the correctness of the models. In this study, an ensemble deep learning algorithm was adopted to extract significant features from MRI scans and feed those features for QML to classify into mild demented, moderated demented, very mild demented, and non-demented. The proposed model detects the AD stages from an MRI of the brain using an ensemble of customized version of VGG16-ResNet50 deep learning models as feature extraction and applying QML algorithms for classification. The suggested approach makes decisions in a more thorough, dependable, and varied manner. The following are the main contributions of our study:

1. The proposed efficient ensemble approach that combines customized version of VGG16 and ResNet50 for Alzheimer's disease feature extraction from ADNI MRI image data
2. We used Quantum Support Vector Machine (QSVM) for classification with high accuracy using ADNI1 and ADNI2 datasets and also explored the effect of quantum ML to effectively improve the computational efficiency of the model
3. We evaluated the proposed models' effectiveness against other cutting-edge techniques. Additionally, we conducted a comparison between classical Support Vector Machine (SVM) and QSVM classifiers.

The order of the remaining sections is as follows. In-depth review of prior works is discussed in Section “Related work”. In Section “Methods”, the background material—which consists of the two primary components, EL and QML—as well as the technique utilized is described. The data set, the models under investigation, and the suggested ensemble model are the main points of interest. The experimental results investigation, acquired data, and model evaluation are presented in Section “Result and discussion”. Section “Conclusion and recommendation” concludes with recommendations for further research.

## Related work

The difficulty of diagnosing AD from MRI scans has been examined, and different approaches have been explored. Modeling the diagnosis as a prediction and classification problem is the most employed approach^9,12,13^. In the light of recent studies Ruiz et al.^13^ proposed an ensemble of 3D densely connected convolutional network models to perform a 4-way classification of 3D MRI images. As every layer in the proposed network is connected to each subsequent layer in a block, dense connections are applied to improve data flow inside the model. Better outcomes come from their studies using the ADNI dataset, which consists of preprocessed 3D MRI images from four subject groups: AD, healthy control, early MCI, and late MCI. Another research study Leela et al.^14^ developed a deep learning-based, automated solution to detect AD early. Durable principal component analysis (RPCA), deep VGG-19 approaches, and the idea of transfer learning were all implemented into their proposed model. The approach plays a role because it is capable of identifying Alzheimer's disease subtypes using fused CT-MRI and EEG signals. Additionally, Chen et al.^6^ introduced Multiview-slice attention and 3D convolution neural network (MSA3D), a fusion model that integrates multiple slice features and 3D structural information organically for AD classification. They fused 2D and 3D features to generate more discriminative representations. As determined by their experimental result, the model obtained accuracy values of 91.1 and 80.1% on ADNI-1 for diagnosing AD and mild cognitive impairment (MCI) convention prediction, respectively. We propose to solve this problem in our research, which is the dearth of data and the inadequate accuracy that still hinders their use in real-life applications. The authors in Kalkan et al.^15^ presented cutting-edge CNN applications using single and multimodality neuroimaging data for AD classification. The authors explored the effective approaches for classifying AD to assess several dataset types, neuroimaging modalities, preprocessing strategies, and data management strategies. CNN has achieved major advances in classifying AD, but there are still many obstacles to overcome, especially given the dearth well neuroimaging data and its potential application in this area. The authors in Amoroso et al.^4^ examined how brain connectivity is affected by AD, using T1 brain Magnetic Resonance Imaging data (MRI) acquired within the ADNI. They showed how graph theory-based models can accurately identify these clinical problems and how game theory's SHapley values applied to make developed models understandable and simple to grasp. The other researchers Orouskhani et al.^16^ introduced a few-shot learning technique called deep metric learning, that utilizes a conditional loss function to overcome the limitation of a few samples and enhance the accuracy of the model. Experiments with OASIS datasets reveal that the model, which was inspired by VGG16, exceeded the most advanced models as a matter of accuracy. Further Baglat et al.^17^ employed diverse machine learning classification algorithms including Logistic Regression, Decision Tree, Random Forest classifier, Support Vector Machine, and AdaBoost for the early identification and classification of Alzheimer ‘s disease using Open Access Series of Imaging Studies (OASIS) dataset. Their efforts revealed to us a noteworthy level of performance and resulted in classification using the Random Forest classifier. The authors Lu et al.^18^ developed an MRI-based AD diagnosis based on deep learning/ transfer learning classifier on significantly vast and diverse datasets. They trained and tested the algorithm on a dataset of unprecedented size and diversity (from more than 217 sites/scan. They constructed an Inception-ResNet-V2 as a sex classifier with high generalization capability and achieved 94.9% accuracy. Another work conducted for the early identification of heart ailments Heidari and Hellstern^10^ presented two quantum machine-learning techniques: a hybrid quantum neural network and a hybrid random forest quantum neural network. Moreover, to estimate the risk of heart disease Abdulsalam et al.^11^ proposed an ensemble machine-learning model based on quantum machine-learning classifiers. The proposed approach used a quantum support vector classifier as the base classifier in a bagging ensemble learning framework. Also, they utilized the SHapley Additive exPlanations (SHAP) framework to figure out and quantify the relevance of each feature in the prediction.

A deep learning pipeline was offered by EL-Geneedy et al.^19^ for comprehensive stage-by-stage classification of Alzheimer's disease (AD). Their approach divides 2D T1 brain MRI images into four stages: very mild dementia, mild dementia, moderate dementia, and non-dementia using a low-level convolutional neural network architecture. The authors conduct a comparative analysis between their methodology and cutting-edge deep learning architectures, such as InceptionV3, DenseNet121, ResNet50, VGG 16, and EfficientNetB7. Reported testing accuracy reached 99.68%. However, further elaboration on the specifics of their model may be necessary.

To classify Alzheimer's disease from cognitively normal and its mild cognitive impairment, Lim and colleagues^20^ developed a model using just three-dimensional brain MRI scans from the ADNI collection. Together with a CNN that was created from scratch, pre-trained VGG-16 and pre-trained ResNet-50 were employed as feature extractors. The most accurate of these models was VGG-16, which had an accuracy of 83.9%.

MRI scans of AD and healthy individuals are similar, making the AD classification task challenging. While the previously stated studies achieved high accuracy when distinguishing between AD and healthy, their practical applicability is still limited because of computationally inefficient classification algorithms and still there is a room for accuracy improvement, which is a limitation that we propose to address in this study. Our method focused on classifying AD dementia stages classification by taking key features based on an ensemble learning model and feeding the combined features to an efficient and robust QSVM classification algorithm.

## Methods

**Figure 1:**
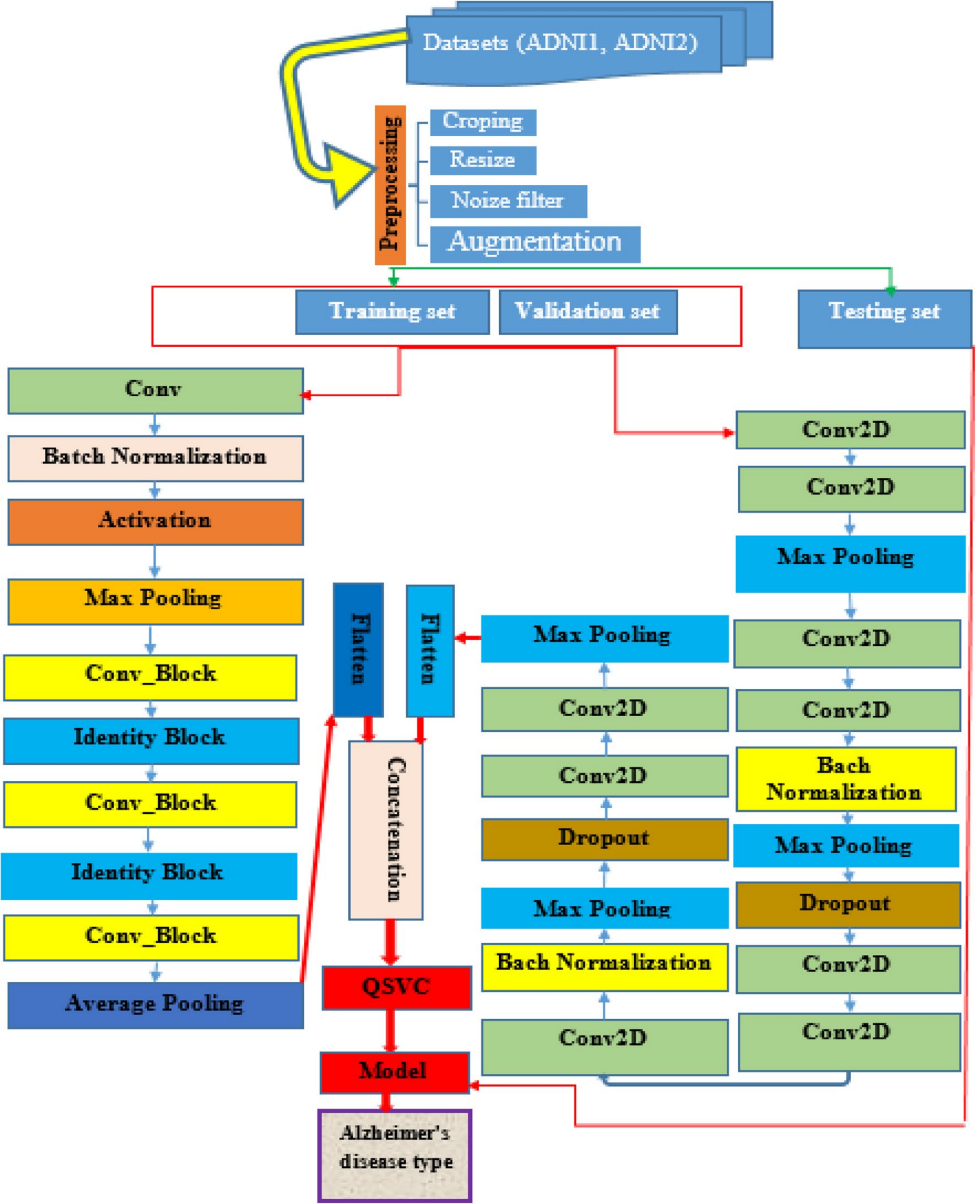
Architecture of the proposed ensemble model with QSVC/QSVM.

The focus of this work is the development of an ensemble DL-based quantum machine learning classification model for the diagnosis of AD disease. Deep learning architectures are the most widely employed for processing and analyzing brain images in research projects^8,18^. In this paper, we introduced a method based on ensemble learning and quantum machine learning classification algorithms that analyze MRI brain images and extract meaningful features for successful classification of AD stages. According to Fig. 1, The proposed algorithm uses sequential steps, The ADNI1 and ADNI2 MRI image data sets are first prepared, pre-processed, and then combined to use in the proposed approach. This is followed by the building of an ensemble model, and its parameters are configured for feature extraction and the features are fed into QSVM for classification of AD dementia stages. Finally, the performance of the proposed model is conducted and compared against the other cutting-edge methods. To judge the models, numerous performance metrics, including accuracy, recall, precision, F1- score, and AUC were calculated. The results implied that the ensemble model-based QSVM is superior to the other cutting-edge methods in terms of performance. Likewise, it can be inferred that effective outcomes can be achieved by combining quantum classifiers and ensemble learning.

As visualized in Fig. 1, the VGGNet model consists nine convolutions, two batch normalizations, three max-pooling, two dropouts, and one flattened layer. A dropout layer is inserted below each max pooling and dense layer to overcome overfitting. while the ResNet model consists of one convolution, one max-pooling, one average polling, one batch normalization, one activation, two identity blocks, three conv blocks, and one flattened layer. Finally, the extracted and flattened features from the two models are concatenated to classify into four Alzheimer's disease types using the QSVM classifier.

### Data and material

The data used to develop the model in our work was retrieved from different sources, such as the ADNI 1 dataset and the ADNI2 dataset from the Kaggle databases **(**ADNI_Extracted_Axial (kaggle.com)**).** Alzheimer's disease Neuroimaging Initiative is a large-scale study focusing on the early detection and progression monitoring of AD. We combine ADNI 1 and ADNI 2 datasets of AD since both are obtained from MRI scans of AD patients in different time stamps. Combining different sources of datasets used to boost deep learning training algorithms for performance improvement. We used only MRI scans of patients from both sources to increase the number of datasets. The high number of datasets helps to reduce overfitting problems in DL algorithms. The summary of our dataset is in Table 1.

**Table 1:** Summary and overview of our datasets.

Diseases	ADNI1	ADNI2	Merged
Mild demented	254	896	1150
Moderated demented	281	64	345
Very mild demented	240	2240	2480
No demented	453	3200	3653

### Preprocessing

The chosen data was pre-processed using a standard processing pipeline. We used a cropping algorithm to eliminate the bone and skeleton portions of the MRI images since this superfluous portion is not significant for AD classification. The original dataset had an image resolution of 176*208. We must scale the MRI image to 128*128 pixels in width and height due to hardware limitations. We lowered the dataset's dimensionality to 5 qubits in order to use it for quantum classification. In our study, we used an adaptive median filter to remove outliers for the facilitation of a reliable classification process. The augmentation technique was applied to increase the number of the Alzheimer’s disease dataset. The efficacy of the augmentation in terms of model over fitting was also improved to increase the generalization capability of deep learning models. Also, there is a class imbalance problem so to alleviate that we used data augmentation comprising arbitrary height and width shift (range, 0–10%) and zooming (range, 0–8%) on the training set.

### Deep learning

Deep learning models, specifically convolutional neural networks (CNN), have revolutionized disease detection in healthcare. We employed the prepared VGG-16 convolutional neural network model, which was enhanced by freezing some of the layers. Simonyan and Zisserman identified the 16-layer convolutional architecture referred to as the VGG-16 model in 2014. The VGG-16 model pertains itself a large network with about 138 million parameters. It piles many convolutional layers to construct deep neural networks that boost their ability to learn hidden features. The network's input image possesses dimensions of (224 × 224 × 3). It additionally includes 16 convolutional layers that work as a fixed size filter (3 × 3) and 5 layers of Max grouping that encompass the entire network in size (2 × 2)^21^. ResNet 50 is perhaps the most powerful convolutional neural network architecture available in the recent decade^22^. It was also selected as the winner of the ILSVRC competition. ResNet-50, a convolutional neural network with 50 layers, is one of the versions of ResNet. A total of 48 convolution layers are included 1 Max pooling and 1 Average pooling layer. It is a deep residual learning framework built on a neural network. It can resolve the vanishing gradient problem even when working with incredibly dense neural networks. ResNet 50, despite the fact it contains 50 layers, has around 23 million trainable parameters, which are significantly less than the trainable parameters of previous architectures. In the residual network rather than learning features, it learns residuals which are the subtraction of learned features from the layer inputs ResNet connects the input of the nth layer directly to an (n + x) th layer, allowing additional layers to be stacked and a deep network established.

#### The proposed ensemble model for feature extraction

The performance of categorizing biomedical signals is enhanced by feature extraction. To increase the efficacy of the classifier, feature extraction intends to discover the most relevant and valuable set of features (unique properties). The most important step in classifying biomedical signals is feature extraction since improperly chosen features could cause the classification performance to suffer^12^. Unlike traditional methods that are time-consuming and require specialized knowledge for feature extraction, deep learning can automatically extract relevant features from input images, resulting in improved prediction accuracy^23^. In this research, we used VGG16- ResNet50 as a base model to exploit the local spatial characteristics of the images. A multitude of features is retrieved from pre-processed image data. The features of MRI images of AD patients are explored by ensemble deep learning models namely customized version of VGG16-ResNet50. Since different CNN architectures can capture diverse information of input images, which increase performance than a single model, the concatenation process of two model features integrates the information from different CNNs to create a more discriminative feature representation than using the feature extracted from a single CNN model^24^. In our Ensemble model, we concatenated the features obtained from the two models as shown in the architecture depicted in Fig. 1.

As previously discussed, the proposed model aims to accurately diagnose AD disease by concatenating deep features extracted from MRI images by using two different models (customized CNN architecture of VGGNet and ResNet50). First, a VGGNet model is proposed to extract features from MRI images. Correspondingly, the ResNet model extracts features from the same images. To end with, the extracted features from these models are flattened and concatenated into a single classification descriptor. Then, the extracted features are fed into the QSVM classifier.

### Classification with quantum machine learning

Support vector machine is a classical machine learning algorithm that uses training data from the sets to classify vectors in a feature space into one of two sets. It attempted to discover a high-probability optimum separation hyperplane between two distinct groups or class in a set of samples, with whole training samples of the class located on one side of the hyperplane. The linear discrimination problem is to develop a hyperplane that may be used as a decision-boundary classification task while also providing substantial differences between two class regions^25^. Quantum-enhanced machine learning techniques can accomplish several tasks, among them lowering training time, managing complex network topology, automatically modifying network hyper parameters, performing complex matrix and tensor manipulation at high speeds, and using quantum tunneling to achieve actual objective function goals, in contrast to traditional machine learning algorithms^26^. In quantum computers, Quantum SVM is the quantum counterpart of the classical SVM. The QSVM has a quantum advantage over the classical SVM in situations where it is challenging to estimate the feature map classically. Using a quantum kernel in QSVM algorithms, quantum computers can accelerate learning by using a quantum kernel^11^. Using quantum feature maps that map data points to quantum states, classical data can be encoded to be processed by a quantum computer^10^. In the following direction, QSVM, a quantum machine learning algorithm was adopted to take the essential features obtained by the ensemble model and classify the MRI scan as AD stages.

**Figure 2:**
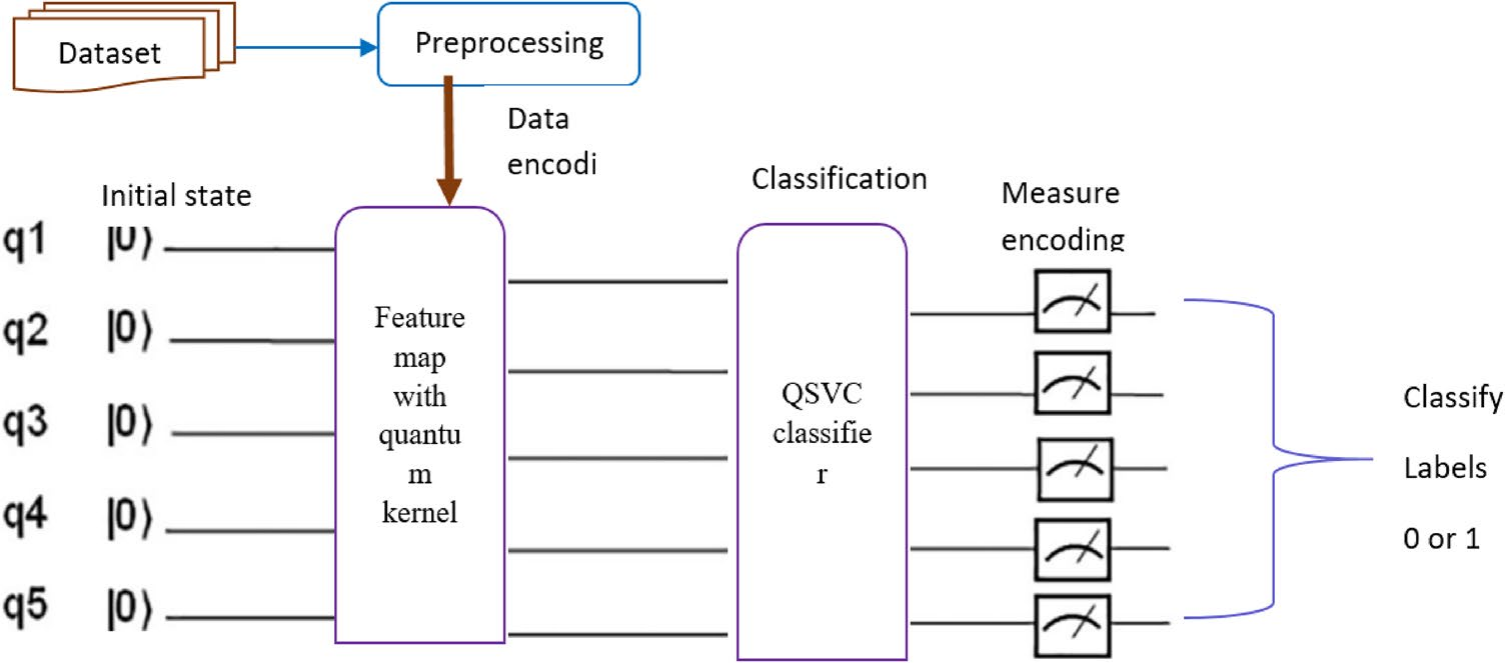
Overview of QSVM.

Figure 2 illustrates the structure of the QSVM algorithm, in which the feature maps are flattened by applying the dense layer. The flattened feature maps were subsequently mapped to quantum spaces using a 5-qubit feature map. By taking the inner product of the quantum feature maps, the quantum kernel maps the quantum state data points into higher-dimensional space. Following the QSVM classifier fitting to the training data and evaluating the performance of the model using the test data, for each classical input, the measurements decode the quantum data into the corresponding classical output data.

## Result and discussion

We conducted the experiments using a Hewlett Packard Core i5, sixth-generation, 8 GB RAM, and a Colab Pro GPU that was manufactured by Google. This section presents all the experiments conducted on four class Alzheimer’s brain disease datasets. We utilized efficient ensemble deep-learning architectures that consumed minimum resources. The parameters utilized for this experiment are presented in Table 2.

**Table 2:** Summary of our proposed model parameter settings.

Batch Size	64
Learning Rate	0.0001
Optimizer	SGD
Loss function	Cross-entropy
No. of Epochs	125
Total Parameters	2,109,079
Trainable Parameters	2,107,927
Non-Trainable Parameters	1,152

### Results of the end-to-end deep learning models

Experiments are conducted on pre trained VGG16 + ResNet50 models but they scored less accuracy in our dataset. This is due to pre trained models are trained using different plants from ImageNet dataset and these image features are different from Alzheimer diseases features that is the reason for these pre trained models scored less accuracy in our experiment. Experiments were conducted using fine-tuned deep learning models including customized version of VGG-16 (VGGNet), ResNet-50 (ResNet), and Ensemble models (VGGNet ResNet).These models were trained and evaluated by applying the categorical cross-entropy loss function for mild demented, moderate demented, non-demented, and very mild demented cases. ResNet and VGGNet were enhanced with a batch normalization layer to speed up training, decrease learning time, and lessen generalization errors. Moreover, a dropout layer was utilized to avoid overfitting. There were 125 epochs implemented for each model. The results of the individual and ensemble models are summarized in Table 3. As compared to individual model VGGNet, ResNet scored the lowest accuracy, precision, recall, F1 score, and area under the curve for Alzheimer’s disease four-class classification. Both the VGG-16 and Ensemble models achieved nearly the same classification accuracy. Ensemble models achieved outstanding performance in individual deep-learning models.

**Table 3:** Results of individual deep learning models.

Model	Accuracy	Precision	Recall	F1-score	AUC
VGGNet	90.11	91.27	89.26	90.23	97.59
ResNet	75.04	79.99	68.37	73.84	93.70
Ensemble model	90.58	92.87	86.14	89.35	98.84

**Figure 3:**
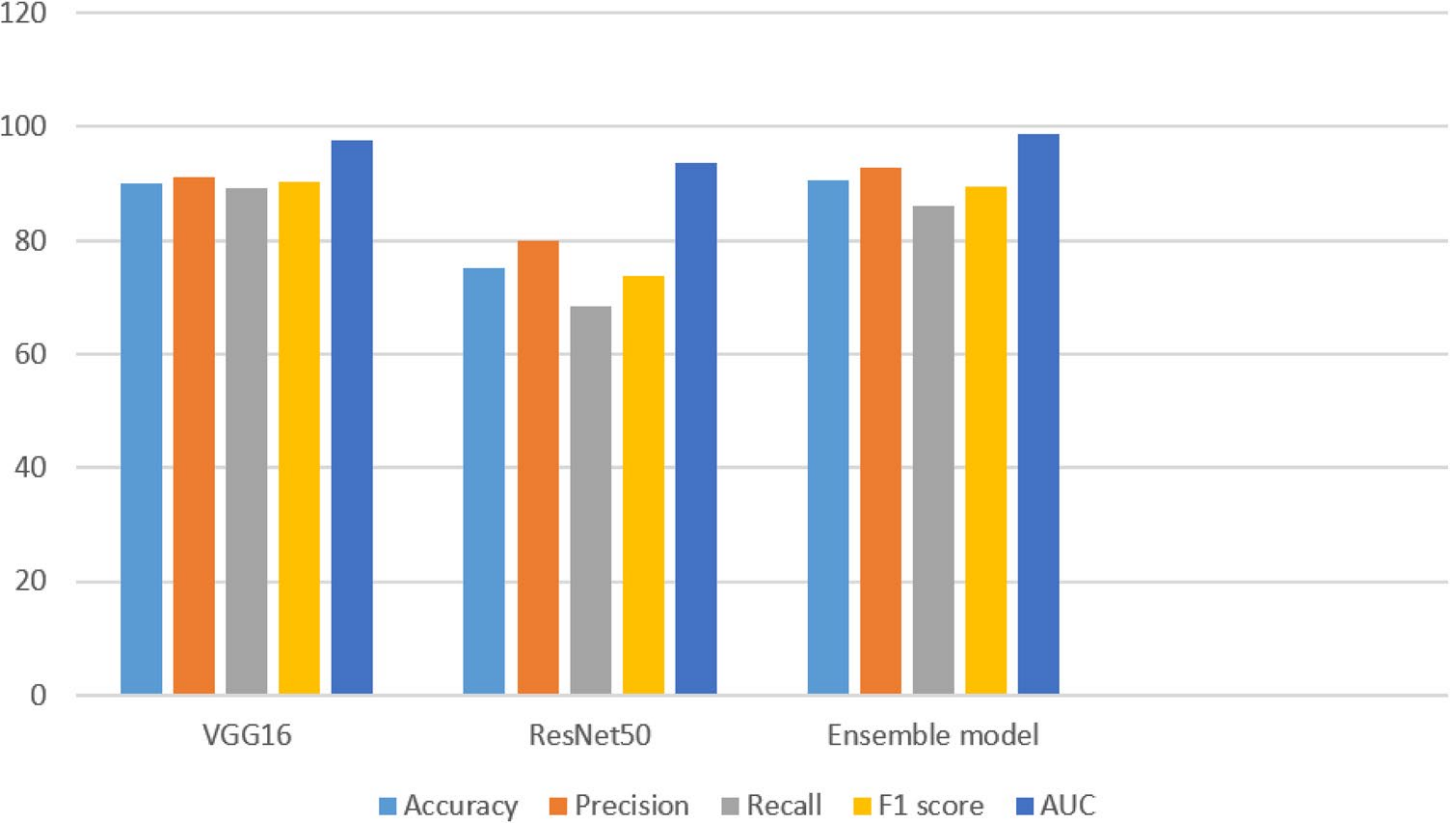
Comparison of individual models using performance metrics.

The performance comparison of individual models using various metrics is shown in Fig. 3. ResNet scored poorly in terms of recall and F1 score. VGGNet achieved outstanding result in addition to ensemble models. When compared to the other metrics, the area under the curve (AUC) fared better.

**Figure 4:**
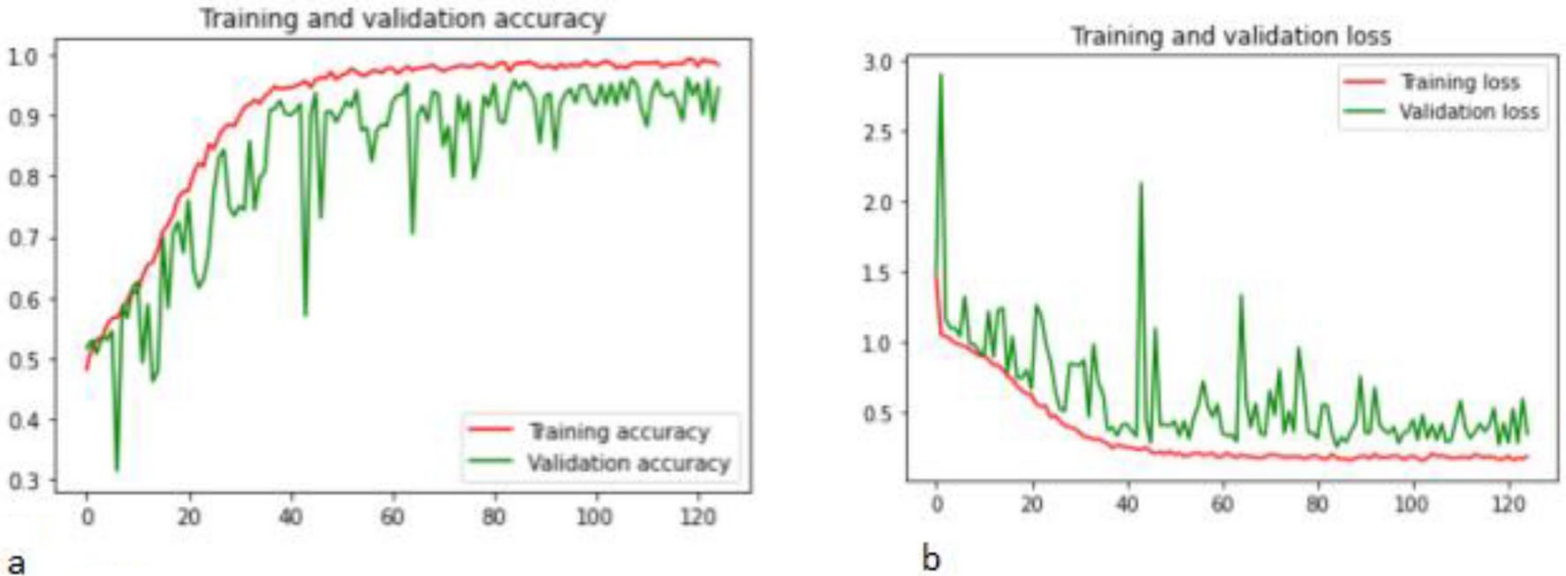
(**a**) training and validation accuracy curves of the proposed ensemble model; (**b**) training and validation loss curves of the proposed ensemble model.

The training-validation accuracy are displayed in Fig. 4a. At epoch 1, we observed that the training accuracy was 52.56, despite this by epoch 10, we started to see disparities in the data. We boosted the performance of the ensemble deep learning models by training them for 125 epochs. Figure 4b presented the performance curves of the ensemble VGGNet + ResNet model, where the training loss was at its lowest point at epoch 80 and the validation loss accuracy at epoch 60. The validation loss dropped off dramatically throughout epochs 40 and also 125.

### The performance of the deep learning models with SVM classification

The effects of the deep models based on SVM were also examined on the Alzheimer’s disease dataset to figure out the effectiveness of the proposed model, as illustrated in Table 4. The VGGNet + SVM model achieved an 85.24% accuracy, 85.00% precision, 85.30% recall, 85% F1 score, and 89.18% area-under-the-curve (AUC) score. The ResNet + SVM model achieved 82.24% accuracy, 85.43 recall, and 84.73 F1-score. The proposed ensemble with the classical SVM model achieved 86.78% accuracy and 90.53% area under the curve (AUC). Even if the proposed ensemble model achieved 86.7% accuracy on the SVM classifier which is better than other models it is not a remarkable result.

**Table 4:** The Performance results of the Deep model with Classical SVM.

Model	Accuracy	Precision	Recall	F1-score	AUC
VGGNet + SVM	85.24	85.00	85.30	85.00	89.18
ResNet + SVM	82.24	82.00	85.43	84.73	85.86
Ensemble model + SVM	86.78	87.77	86.98	87.54	90.53

### The performance of the deep learning models with QSVM classification

The results of our proposed ensemble model, deep learning models with QSVM classifier are presented in Table 5. The VGGNet with QSVM achieved a 95.65% accuracy and 98.85% AUC. ResNet + QSVM achieved a 91.56% accuracy and 97.87% AUC. The proposed VGG-Net + ResNet + QSVM model achieved a 99.89% accuracy score and a 99.99% area under the curve (AUC). All the ensemble models achieved good performance results and accurately classify the AD stages from the merged ADNI dataset. The ResNet + QSVM model achieved 6% better accuracy than the standalone ResNet end-to-end model. The Proposed model achieved 8.5% and 12.21% better results when we compared it with the proposed end-to-end ensemble model and ensemble with SVM respectively.

**Table 5:** Results of deep learning models with QSVM.

Model	Accuracy	Precision	Recall	F1-score	AUC
VGGNet + QSVM	95.65	95.49	95.32	95.45	98.85
ResNet + QSVM	91.56	91.69	91.12	90.95	97.87
The proposed ensemble model + QSVM	99.89	99.25	99.23	99.13	99.99

**Figure 5:**
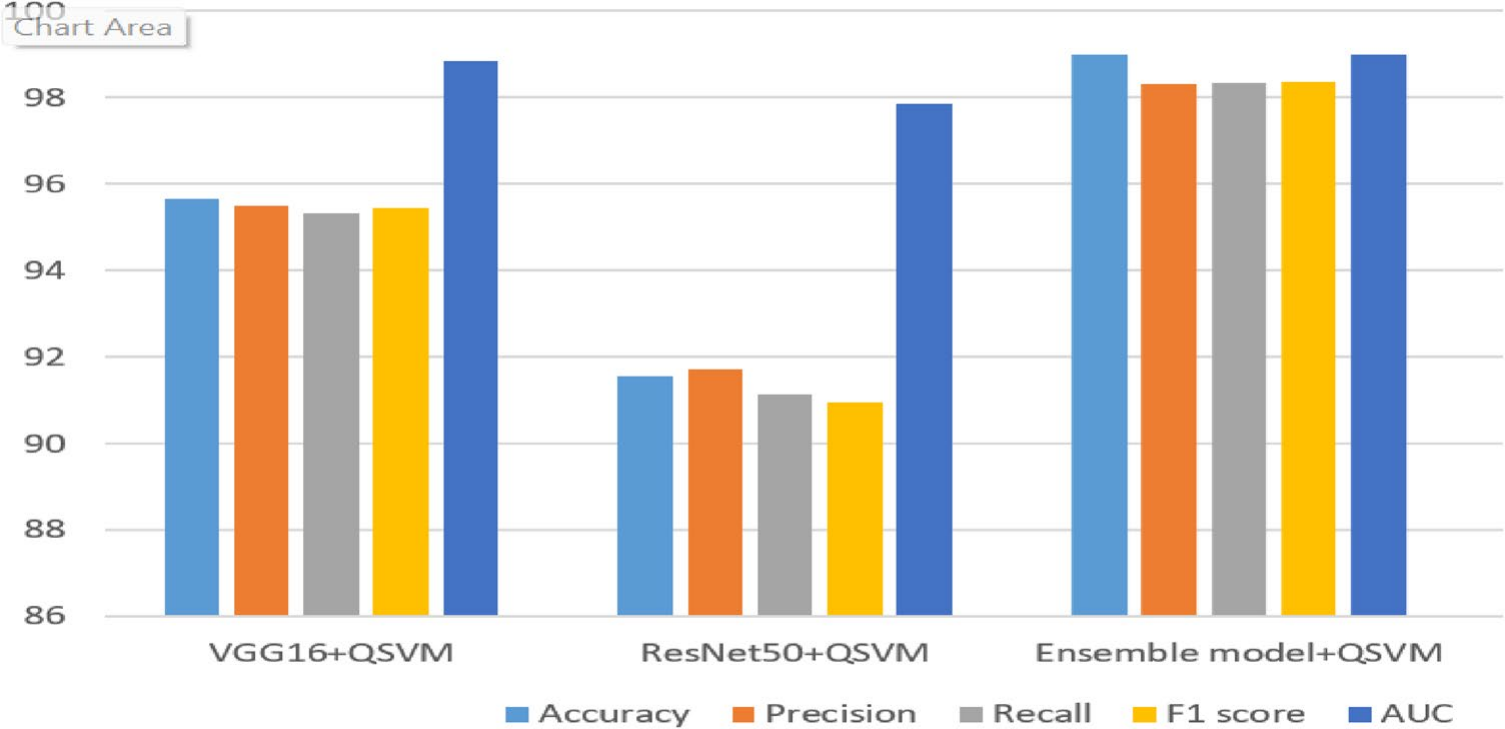
Comparison of deep models using performance metrics with QSVM.

The performance comparison of the ensemble models with other models is presented in Fig. 5. Among the ensemble models with other deep models, the proposed ensemble model performed efficiently, with high-performance metrics. The VGGNet with QSVM achieved outstanding results than the end-to-end VGGNet and VGGNet + SVM. Similarly, ResNet with QSVM scored with remarkable accuracy as compared to end-to-end ResNet and ResNet with SVM. The experiments validate that using QSVM for classification provided excellent results in all deep learning models in all evaluation metrics. Table 6 tabulates a systematic comparison of the present work with previous related approaches.

**Table 6:** Comparison of the present work with previous architectures.

References	Method	Dataset	Accuracy (%)
El-Geneedy et al.^19^	CNN	OASIS	99.68
Lim et al.^20^	VGG 16	ADNI	83.9
Lu et al.^18^	Inception-ResNet-V2	ADNI and unseen independent datasets (AIBL,MIRIAD,OASIS)	94.9
P. et al.^27^	AlexNet	ADNI	93.24
Baglat et al.^17^	Random Forest and Adaptive boosting classifier	OASIS	86
Proposed	VGGNet + ResNet + QSVM	ADNI1 + ADNI2	99.89

## Conclusion and recommendation

The challenge of AD classification pushed us to develop an efficient model by integrating different source data to come up with a better outcome prediction. This paper proposed an ensemble DL model with QSVM, for AD classification that utilizes the learned features to feed into the QSVM. Using the ADNI1 and ADNI2 MRI data taken from Kaggle, we evaluated the performance of ensemble learning methods with the classical SVM and QSVM classifiers. We used 5 qubit quantum hardware or simulator and we utilized the QSVM model from the Qiskit library and optimized it by adding the hyper parameters. According to the experimental results, the proposed model outperforms the classical SVM in terms of AD classification accuracy and training time. It provides a significant solution to support AD primary care, especially where the MRI scan is blurred and difficult for experts to properly suggest the disease. However, further research needs to be conducted to evaluate implementation scenarios by integrating the model within medical devices for AD diagnosis.

## Data Availability

Data Availability In this article, the ADNI dataset are used which can be found from the address https://www.kaggle.com/datasets/tourist55/alzheimers-dataset-4-class-of-images”.
